# Polycystic Ovary Syndrome and Risk of Five Common Psychiatric Disorders Among European Women: A Two-Sample Mendelian Randomization Study

**DOI:** 10.3389/fgene.2021.689897

**Published:** 2021-06-15

**Authors:** Luyang Jin, Jia'en Yu, Yuxiao Chen, Haiyan Pang, Jianzhong Sheng, Hefeng Huang

**Affiliations:** ^1^Department of Reproductive Endocrinology, Women's Hospital, School of Medicine, Zhejiang University, Hangzhou, China; ^2^Key Laboratory of Reproductive Genetics, Ministry of Education, School of Medicine, Zhejiang University, Hangzhou, China; ^3^The First Affiliated Hospital, School of Medicine, Zhejiang University, Hangzhou, China; ^4^Department of Pathology and Pathophysiology, School of Basic Medical Sciences, Zhejiang University, Hangzhou, China

**Keywords:** Mendelian randomization, polycystic ovary syndrome, psychiatric disorders, causality, single nucleotide polymorphism

## Abstract

**Background:** Observational studies have implied an association between polycystic ovary syndrome (PCOS) and psychiatric disorders. Here we examined whether PCOS might contribute causally to such disorders, focusing on anxiety disorder (AD), bipolar disorder (BIP), major depression disorder (MDD), obsessive compulsive disorder (OCD), and schizophrenia (SCZ).

**Methods:** Causality was explored using two-sample Mendelian randomization (MR) with genetic variants as instrumental variables. The genetic variants were from summary data of genome-wide association studies in European populations. First, potential causal effects of PCOS on each psychiatric disorder were evaluated, and then potential reverse causality was also assessed once PCOS was found to be causally associated with any psychiatric disorder. Causal effects were explored using inverse variance weighting, MR-Egger analysis, simulation extrapolation, and weighted median analysis.

**Results:** Genetically predicted PCOS was positively associated with OCD based on inverse variance weighting (OR 1.339, 95% CI 1.083–1.657, *p* = 0.007), simulation extrapolation (OR 1.382, 95% CI 1.149–1.662, *p* = 0.009) and weighted median analysis (OR 1.493, 95% CI 1.145–1.946, *p* = 0.003). However, genetically predicted OCD was not associated with PCOS. Genetically predicted PCOS did not exert causal effects on AD, BIP, MDD, or SCZ.

**Conclusions:** In European populations, PCOS may be a causal factor in OCD, but not AD, BIP, MDD, or SCZ.

## Introduction

Polycystic ovary syndrome (PCOS) affects 5–15% of women of reproductive age, making it one of the most common endocrine disorders in that group. It is characterized mainly by hirsutism, elevated serum testosterone, oligo-/amenorrhea, and polycystic ovaries (Rosenfield and Ehrmann, 2016). Because of its high prevalence and association with infertility and long-term metabolic complications (diabetes, cardiovascular disease), PCOS is a major threat to women's health and a burden on the economy in the amount of 4.36 billion USD annually (Azziz et al., 2005).

Several studies have revealed a relationship between PCOS and mental illness. For instance, women with PCOS are at increased risk for psychiatric disorders, including anxiety disorder (AD), bipolar disorder (BIP), major depression disorder (MDD), obsessive compulsive disorder (OCD), and schizophrenia (SCZ) (Hung et al., 2014; Blay et al., 2016; Cesta et al., 2016; Brutocao et al., 2018). PCOS is a lifelong morbidity, and the onset of mental illness at any time in a women's life can further aggravate her disease burden. Therefore, early detection of psychiatric disorders in women with PCOS is important in order to enable timely intervention to prevent progression or recurrence of such disorders.

Whether the association between PCOS and psychiatric disorders reflects a causal relationship is unclear. This is due, in part, to the heterogeneity of PCOS and the presence of unavoidable confounders, including obesity, hyperandrogenism, insulin resistance, and inflammation. A method for exploring such potential causality in an unbiased way is two-sample Mendelian randomization (MR), which relies on genetic variants as instrumental variables (IVs) to evaluate causality between an exposure and an outcome (Davies et al., 2018). After random allocation during meiosis, single-nucleotide polymorphisms (SNPs) remain stable and unmodified throughout a lifetime of environmental exposure, making them independent of confounders or reverse causation (Sekula et al., 2016). MR has been already proven powerful for resolving controversies about PCOS: while epidemiologic studies suggested that women with PCOS were more likely to experience type 2 diabetes mellitus, coronary heart disease, or stroke, MR detected no genetic causality between these diseases and PCOS (Zhu et al., 2020). MR has also suggested that PCOS is positively associated with breast cancer (Wen et al., 2020) and negatively associated with ovarian cancer (Harris et al., 2019), despite earlier studies showing no such associations (Barry et al., 2014).

Therefore, we applied MR analysis to summary data from European genome-wide association studies (GWAS) in order to examine potential causal associations of PCOS with five common psychiatric disorders: AD, BIP, MDD, OCD, and SCZ.

## Materials and Methods

### Data

GWAS data for PCOS were taken from Apollo (https://doi.org/10.17863/CAM.27720), while GWAS data for AD, BIP, MDD, OCD, and SCZ were taken from the Psychiatric Genomics Consortium (https://www.med.unc.edu/pgc/results-and-downloads) (Table 1). All subsequent analyses were restricted to “European” as the ethnic cohort. The ID of SNPs, the associated effect size (β), standard error (se) of the effect size, and effect allele were extracted from each GWAS summary dataset.

**Table 1:** Overview of genome-wide association datasets for polycystic ovary syndrome and five psychiatric disorders.

**Disease or trait**	**File name**	**Number of cases**	**Number of controls**	**PMID**
Polycystic ovary syndrome	PCOS _19092018	10,074	103,164	30566500
Anxiety disorder	ANGST	7,016	14,745	26754954
Bipolar disorder	BIP 2018	20,352	31,358	31043756
Major depression disorder	PGC MDD No UKB/No 23 and Me	45,396	97,250	29700475
Obsessive compulsive disorder	OCD	2,088	7,037	28761083
Schizophrenia	SCZ EUR	33,640	43,456	31740837

### Estimation of Participant Overlap

The original articles that published GWAS exposure and outcome data were obtained to retrieve detailed information about study design and sample collection. Participants recruited from the same consortium or hospital in the exposure and outcome studies were regarded as duplicates. The maximized overlap was calculated as n/N, where n referred to the number of potentially repetitive recruiters, and N to the total sample number in the larger dataset (Burgess et al., 2016).

### Selection of IVs

Several quality control steps were conducted to select eligible SNPs as IVs from the exposure data. First, SNPs had to meet genome-wide significance, defined as *p* < 5 × 10^−8^. Second, the SNPs had to be independent of each other, so clumping (criteria: *r*^2^ = 0.001, kb = 10,000) was performed to exclude linkage disequilibrium (LD) between the SNPs. Among SNP-shaving LD, only those with the lowest *p*-values were retained. Third, SNPs related to a confounder-associated phenotype were removed. The potentially related phenotypes were detected using the online database “PhenoScanner” (www.phenoscanner.medschl.cam.ac.uk/phenoscanner), by filtration of *r*^2^ > 0.8 and *p* < 5 × 10^−8^.

### Two-Sample MR Analysis

Before statistical analysis of causal relationships, we ensured that GWAS exposure and outcome data matched well. First, information about exposure-associated SNPs was extracted from each outcome dataset. Any exposure-associated SNP absent from the outcome dataset was substituted with a proxy SNP; that is, if it existed, and it was in LD (*r*^2^ > 0.8, MAF for palindromes <0.3) with the requested one. Second, the exposure and outcome data were harmonized to ensure that the effect of the SNP was on the same allele. Otherwise, the SNP was deleted.

Third, the statistical significance of the matched exposure-outcome summary data was analyzed by several methods. Inverse variance weighted (IVW) analysis assumed no or balanced pleiotropy. We used the random-effects model to avoid heterogeneity bias, which was measured using Cochran's Q test. The MR-Egger method not only allowed for, but also detected, horizontal pleiotropy based on its intercept with a y-axis. When the intercept was not zero, there was horizontal pleiotropy. The MR-Egger method was based on the “instrument strength independent of the direct effects” (INSIDE) and “no measurement error in the SNP exposure effects” (NOME) assumptions, and it also used the random-effects model. In addition, when the regression dilution *I*^2^ statistic was <90%, indicating violation with the NOME assumption, the simulation extrapolation (SIMEX) correction was performed (Bowden et al., 2016). The WM method was used to generate unbiased results when ≥50% SNPs were valid variants.

Fourth, SNP pleiotropy and sensitivity were assessed using pleiotropy tests, forest plots, funnel plots, and leave-one-out plots. The forest plot estimated the causal effect of each SNP on the outcome by using Wald ratio analysis. The Funnel plot was used to assess heterogeneity by depicting the reciprocal of the se of the SNP against SNP effects on the outcome. The leave-one-out plot ascertained whether an association was disproportionately influenced by a single SNP. In such a plot, each black point represented the IVW analysis after exclusion of that particular SNP.

### Forward and Reverse Two-Sample MR Analyses

We investigated whether PCOS-SNPs could induce major mental illness (AD, BIP, MDD, OCD, and SCZ), so the direction of MR analysis was defined as forward when PCOS was the exposure and psychiatric disorders were the outcomes. When PCOS was found to be causally associated with any outcome, reverse MR analysis was performed, in which the precise psychiatric disorder was the exposure and PCOS was the outcome. This allowed us to eliminate bias due to reverse causation.

### Statistical Analysis and Multiple Testing Correction

Analyses were conducted using the “sqldf,” “biomaRt,” “TwoSampleMR,” “MendelianRandomization,” or “simex” modules in the R package (version 4.0.3). In general, statistical significance was defined as *p* < 0.05. In the case of multiple testing, the threshold for statistical significance was adjusted by the conservative Bonferroni correction according to the formula *p* < 0.05/n, where *n* referred to the number of MR tests. Since the present study included five forward and one reverse two-sample MR analyses, the adjusted *p*-values for the forward and reverse MR tests were, respectively, 0.01 (0.05/5) and 0.05 (0.05/1). When a *p*-value for an association was below the adjusted cut-off value, the association was considered statistically significant as a causal association. When a *p*-value was below 0.05 but equal to or greater than the adjusted cut-off value, the association was considered one of potential causality needing further confirmation. When a *p*-value was equal to or >0.05, it was considered not to be causal.

### Bioethics

Ethics approval was not required since the data were published GWAS summary statistics from public databases.

## Results

### Participant Overlap Between PCOS and Each Psychiatric Disorder

Two-sample MR analysis requires that the exposure and outcome studies be conducted in two independent, non-overlapping cohorts from the same population. High participant overlap can increase type I error, biasing the MR results (Burgess et al., 2016). The estimated participant overlap between PCOS and each psychiatric disorder was low: 5.8028% for AD, 0.2296% for BIP, 5.2101% for MDD, 0% for OCD, and 2.6174% for SCZ (Table 2). We considered such low overlap as unlikely to bias subsequent analyses.

**Table 2:** Participant overlap between polycystic ovary syndrome and each psychiatric disorder.

**Psychiatric disorder**	**Potential overlapped participants with PCOS**		**Overlap rate**
	**Cases**	**Controls**	
Anxiety disorder	1,112	5,459	5.8028%
Bipolar disorder	130	130	0.2296%
Major depression disorder	658	6,774	5.2101%
Obsessive compulsive disorder	0	0	0%
Schizophrenia	157	2,807	2.6174%

### IVs for PCOS

Among the most significant 10,000 SNPs of the largest GWAS meta-analysis in European women with PCOS (Supplementary Material), 14 remained after filtering for genome-wide significance (*p* < 5 × 10^−8^) and eliminating SNPs in LD (Table 3). Then whether these 14 SNPs had other confounder-associated phenotypes was detected. Four SNPs were found to be related to other phenotypes: rs11031005, rs13164856, rs7864171, rs2271194. The first three SNPs were related to irrelevant secondary phenotypes. Although rs2271194 was related to body mass index and asthma, the effect sizes of rs2271194 on body mass index (β = 0.01348) and asthma (0.005336) were too small to bias the results. Hence none of them were excluded and the final set of PCOS-IVs contained 14 SNPs (Table 3).

**Table 3:** The 14 single-nucleotide polymorphisms (SNPs) used as instrumental variables (IVs) of polycystic ovary syndrome.

**rsID**	**Nearest gene**	**Effect allele**	***β***	**se**	***p*-value**	Secondary phenotype^*^
rs2178575	ERBB4	A	0.1663	0.0219	3.34E-14	/
rs11031005	FSHB	T	−0.1593	0.0223	8.66E-13	Length of menstrual cycle Age at menopause Years since last cervical smear test Bilateral oophorectomy Irregular menstruation
rs804279	NEIL2	A	0.1276	0.0184	3.76E-12	/
rs11225154	YAP1	A	0.1787	0.0272	5.44E-11	/
rs9696009	DENND1A	A	0.202	0.0311	7.96E-11	/
rs13164856	IRF1-AS1	T	0.1235	0.0193	1.45E-10	Sum eosinophil and basophil counts Eosinophil count Height Neutrophil percentage of granulocytes Platelet count Whole body water mass Sitting height
rs1784692	ZBTB16	T	0.1438	0.0226	1.88E-10	/
rs7563201	THADA	A	−0.1081	0.0172	3.68E-10	/
rs8043701	CASC22	A	−0.1273	0.0208	9.61E-10	/
rs1795379	LOC105369844	T	−0.1174	0.0195	1.81E-09	/
rs853854	MAPRE1	A	−0.0975	0.0163	2.36E-09	/
rs10739076	PLGRKT	A	0.1097	0.0197	2.51E-08	/
rs7864171	C9orf3	A	−0.0933	0.0168	2.95E-08	Height Trunk fat-free mass
rs2271194	ERBB3	A	0.0971	0.0166	4.57E-09	Allergic disease Eosinophil count Asthma Whole body water mass Rheumatoid arthritis Years of educational attainment Body mass index

* *Secondary phenotypes were detected by PhenoScanner (www.phenoscanner.medschl.cam.ac.uk), with filtration of p <5 × 10^−8^ and r^2^ > 0.8*.

*rsID, the ID of the single nucleotide polymorphism (SNP); β, the effect size of the SNP; se, the standard error of the effect size; chr, chromosome; posi, position*.

### Causal Effect From PCOS to Psychiatric Disorders

**Figure 1:**
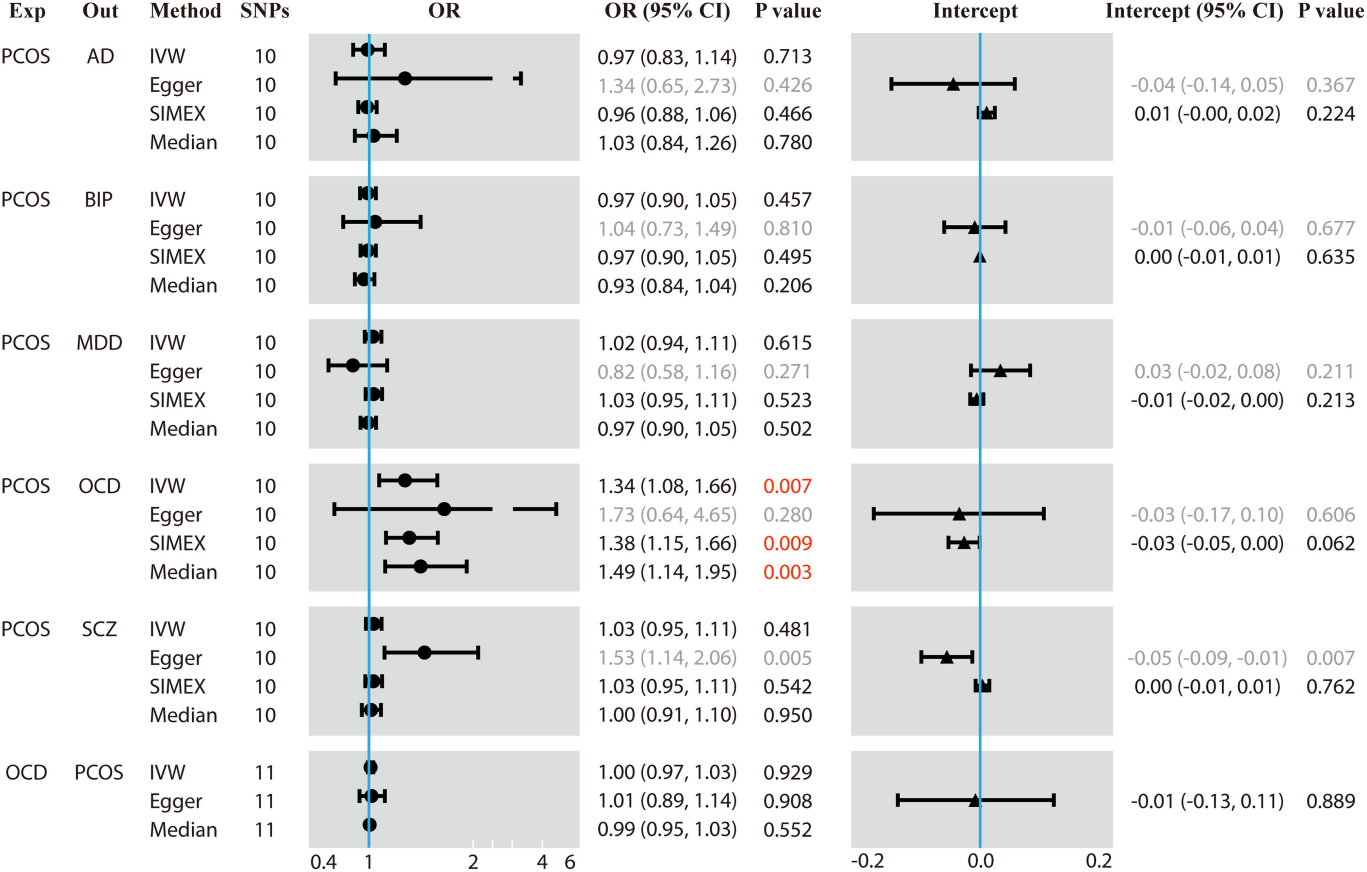
Results of forward and reverse Mendelian randomization (MR) analyses. Two-sample MR analysis showing the effect of the exposure on the outcome using single-nucleotide polymorphisms (SNPs) significant at *p* < 5 × 10^−8^. Exp, exposure; Out, outcome; SNPs, number of the SNPs used in MR analysis; OR, odds ratio; CI, confidence interval; PCOS, polycystic ovary syndrome; AD, anxiety disorder; BIP, bipolar disorder; MDD, major depression disorder; OCD, obsessive compulsive disorder; SCZ, schizophrenia. The regression dilution *I*^2^ statistics between PCOS exposure and each of the five psychiatric disorders were <90%, which made the results of the Egger method inaccurate. Therefore, simulation extrapolation (SIMEX) correction was applied. OR values are binary, while the intercept values are continuous. Values in red mean that they are statistically significant.

**Figure 2:**
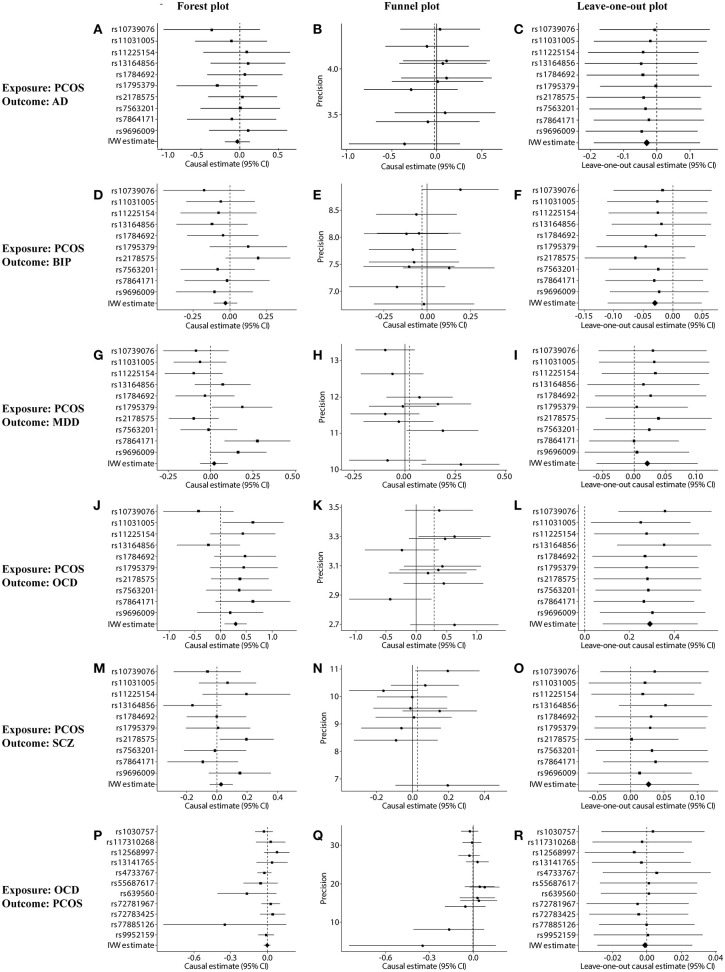
Sensitivity tests of forward and reverse Mendelian randomization (MR) analyses. **(A–O)** Forest, funnel, and leave-one-out plots for MR analysis between PCOS (exposure) and one of the following outcomes: **(A–C)** AD, **(D–F)** BIP, **(G–I)** MDD, **(J–L)** OCD, or **(M–O)** SCZ. **(P–R)** Forest, funnel, and leave-one-out plots for MR analysis between OCD (exposure) and PCOS (outcome). Plots were generated based on the effect size of the SNP (beta) and its standard error (se). PCOS, polycystic ovary syndrome; AD, anxiety disorder; BIP, bipolar disorder; MDD, major depression disorder; OCD, obsessive compulsive disorder; SCZ, schizophrenia.

During data harmonization before MR, rs804279, rs8043701, rs853854, and rs2271194 were discarded because they were found to be palindromic SNPs with intermediate allele frequencies. MR analyses involving different methods are detailed in Figure 1, and the sensitivity analyses are displayed in Figure 2. The *I*^2^ statistic determined by the MR-Egger method in each MR was <90%, suggesting NOME violation and regression dilution, which can inflate type I error (Bowden et al., 2016). Therefore, the results from the MR-Egger method were considered inaccurate and therefore corrected with the SIMEX method (Bowden et al., 2016).

Genetically predicted PCOS was positively associated with OCD according to models based on random-effect IVW (OR 1.339, 95% CI 1.083–1.657, *p* = 0.007), SIMEX (OR 1.382, 95% CI 1.149–1.662, *p* = 0.009) and WM (OR 1.493, 95% CI 1.145–1.946, *p* = 0.003). The causal estimates from the three methods were consistent in direction and magnitude, which is unlikely to be a coincidence. In addition, no significant heterogeneity was detected by Cochran's Q statistic in the IVW analysis (3.2554, *p* = 0.3308) or MR-Egger analysis (2.4418, *p* = 0.2708), and no significant horizontal pleiotropy was detected based on the SIMEX intercept (β = −0.027, *p* = 0.062). The funnel plot showed symmetric distribution, further validating the absence of heterogeneity. The leave-one-out plot showed that excluding any single SNP from the IVs did not substantially alter the results. This suggests that the association between PCOS and OCD was not driven by any single SNP and is therefore likely to be reliable.

In contrast, genetically predicted PCOS was not related to AD, BIP, MDD or SCZ. The results from all three types of MR analyses were consistent, no significant heterogeneity or horizontal pleiotropy was observed, and no single SNP strongly drove the overall effect. Hence, these negative associations are likely to be reliable.

### Causal Effect From OCD to PCOS

To eliminate the bias of reverse causation, the causal effect from OCD to PCOS was assessed (Figures 1, 2). No SNPs were found to be associated with OCD with genome-wide significance (*p* < 5 × 10^−8^), so we relaxed the threshold to *p* < 5 × 10^−6^, leading to a set of 15 independent SNPs as IVs (Table 4). The SNPs rs1163477, rs1256899, rs5634380, and rs909701 were discarded during data harmonization because they were found to be palindromic SNPs with intermediate allele frequencies. The *I*^2^ of the MR-Egger method was 98.8% (>90%), so the results were not corrected using SIMEX.

**Table 4:** The 15 single-nucleotide polymorphisms (SNPs) used as instrumental variables of obsessive compulsive disorder (OCD).

**rsID**	**Nearest gene**	**Effect allele**	***β***	**se**	***p*-value**	Secondary phenotype^*^
rs1030757	GRID2	A	0.847639	0.0339	1.09E-06	/
rs116347760	MAGI3	A	1.87705	0.1335	2.39E-06	/
rs117310268	RP11-595B24.2	T	1.56627	0.0965	3.31E-06	/
rs12504244	NMU	C	0.844593	0.0352	1.62E-06	Myeloid white cell count
rs12568997	RN7SL583P	A	0.745798	0.058	4.23E-07	/
rs13141765	RP11-586D19.1	T	0.766209	0.0558	1.86E-06	/
rs4733767	CASC8	A	1.21349	0.039	7.10E-07	/
rs55687617	RP11-10F11.3	A	0.763227	0.0575	2.67E-06	/
rs56343802	AHCYP4	A	0.843834	0.0368	3.97E-06	/
rs639560	PLA2G4D	T	0.659812	0.0879	2.23E-06	/
rs72781967	RP13-463N16.6	T	0.846538	0.0353	2.43E-06	/
rs72783425	MKL2	A	1.40453	0.0733	3.53E-06	/
rs77885126	RP11-325K19.1	T	0.547441	0.1312	4.38E-06	/
rs909701	LINC00207	C	0.856073	0.0337	4.05E-06	/
rs9952159	DLGAP1	T	1.19997	0.0396	4.21E-06	/

* *Secondary phenotypes were detected by PhenoScanner (www.phenoscanner.medschl.cam.ac.uk), with filtration of p < 5 × 10^−8^ and r^2^ > 0.8*.

*rsID, the ID of the single nucleotide polymorphism (SNP); β, the effect size of the SNP; se, the standard error of the effect size*.

Genetically predicted OCD showed no association with PCOS based on models involving random-effect IVW (OR 0.999, 95% CI 0.971–1.026, *p* = 0.929), MR-Egger (OR 1.007, 95% CI 0.890–1.139, *p* = 0.908) or WM (OR 0.989, 95% CI 0.953–1.026, *p* = 0.552). We found no evidence of significant heterogeneity or horizontal pleiotropy that might bias the results, and no single SNP strongly drove the overall effect. We conclude that the relationship between PCOS and OCD is not likely to be biased by reverse causation.

## Discussion

In this study, summary GWAS data from European populations were used to investigate the potential causality between PCOS and five common psychiatric disorders. In our analysis, genetically predicted PCOS increased the risk for OCD, but it was not related to AD, BIP, MDD, and SCZ.

Several factors lead us to consider our results as likely to be reliable. First, participant overlap, which would increase false positives, was rather low, ranging from 0 to 5.8028%. The SNPs from PCOS-IVs were strongly associated with PCOS, reflected in *F* statistic values >30 (Zhu et al., 2020), much higher than the cut-off of >10 for a strong association (Pierce et al., 2011). This should help reduce bias. In fact, using the metric of Burgess et al. (2016), in which the overlap rate is divided by the F statistic, we estimate that the relative bias in the present study ranged from 0 to 0.1934%, since our maximal overlap rate was 5.8028% and the minimal *F* statistic was 30. This implies minimal bias in our MR results. Second, our IVs satisfied all three assumptions needed for MR analysis (Emdin et al., 2017): all IVs were closely associated with the exposure (“relevance assumption”), they were not associated with any other risk factors (“independence assumption”), and the IVs affected the outcome exclusively through exposure (“exclusion restriction assumption”). Third, the three types of MR analysis gave consistent causal estimates, further implying reliability. Nevertheless, due to the complexity of PCOS and psychiatric disorders, further investigations are still needed to confirm our results.

Our results showed that the genetically predicted PCOS positively associated with OCD, whereas the genetically predicted OCD was irrelevant to PCOS, confirming that the genetic causal direction was from PCOS to OCD. Consistent with our results, a previous study found that women with PCOS were more susceptible to OCD (OR 1.37, 95% CI 1.22–1.55) and their OCD symptoms were more severe (Brutocao et al., 2018). Similarly, a cross-sectional study of an Australian population showed increased prevalence of OCD among women with PCOS (adjusted OR 1.8, 95% CI 1.2–2.5) (Tay et al., 2020). A study of an Indian population found OCD prevalence of 6.36% among women with PCOS and of 2.5% among controls (Hussain et al., 2015).

Although PCOS regarded as an ovarian disease, the aberrant brain circuits concerning hyperactive gonadotropin-releasing hormone (GnRH), highlights the neuroendocrinal role in PCOS etiology (Moore and Campbell, 2017; Ruddenklau and Campbell, 2019). The GnRH neurons, located dispersedly in the rostral forebrain, receive inputs from upstream afferent neurons and regulate the downstream pulsatile release of luteinizing hormone (LH) in anterior pituitary. The gamma-Aminobutyric acid (GABA) and kisspeptin/neurokinin B/dynorphin (KNDy) neurons in the arcuate nucleus are two important afferent neurons (Ozgen and Yildiz, 2021). In prenatally androgenized (PNA) PCOS-like mice, the GABA neuron axon inputs and GABAergic post-synaptic currents to GnRH neurons were increased, causing hyperactive GnRH impulses (Moore et al., 2015; Coutinho and Kauffman, 2019). The kisspeptin and neurokinin B (NKB) signaling have been found to increase GnRH release (Terasawa et al., 2018; Coutinho and Kauffman, 2019). In PNA PCOS-like rats, the number of kisspeptin- and NKB-positive cells, as well as the expression of Kiss1 (gene for kisspeptin) and Tac2 (gene for NKB), were increased in arcuate nucleus (Yan et al., 2014; Osuka et al., 2017), which possibly increased GnRH-neuron activity.

How PCOS may contribute to OCD is unknown. In fact, little is known about how OCD occurs. It is a chronic, debilitating psychiatric illness, characterized by obsessions (intrusive and disturbing thoughts), compulsions (repetitive behaviors), or both. OCD appears to involve the cortico-striato-thalamo-cortical (CSTC) circuit, as well as the fronto-limbic, fronto-parietal, and cerebellar networks (Stein et al., 2019; Bellia et al., 2021). Within the CSTC circuit, defects in neurotransmission involving serotonin, catecholamine/dopamine and glutamate appear to contribute to OCD (Stein et al., 2019). Indeed, several genetic polymorphisms have been linked to OCD involving serotonin signaling (in the genes SLC6A4, HTR2A, HTR2C, HTR1B, TPH1, TPH2), catecholamine/dopamine system (COMT, SLC6A3, MAOA, DRD2, DRD3, DRD4), and glutamate system (SLC1A1, GRIN2B, GRIK2, GRIK3) (Bellia et al., 2021).

Some SNPs among the PCOS-IVs in the present study play roles in neurotransmitter systems. For instance, rs2178575 lies in an intron of the gene encoding Erb-B2 receptor tyrosine kinase 4 (ERBB4), which is expressed in GABAergic interneurons but also in midbrain dopaminergic neurons, where it regulates dopamine levels by binding to neuregulin 1 (NRG1) (Skirzewski et al., 2020). Mice lacking ErbB4 show regional imbalances in basal dopamine levels and fail to increase dopamine release in response to local NRG1 infusion in the dorsal hippocampus, medial pre-frontal cortex and dorsal striatum (Skirzewski et al., 2018). The SNP rs11225154 also lies in an intron of the gene encoding Yes1-associated transcriptional regulator (YAP1), which promotes survival of dopaminergic neurons by reducing the synthesis of the apoptotic protein PTEN (Zhang et al., 2017). The SNP rs10739076 lies in an intron upstream of plasminogen receptor with a C-terminal lysine (PLGRKT). This protein is expressed on the surface of catecholaminergic cells, where it stimulates plasminogen activation and regulates catecholamine release (Bai et al., 2011). Future studies should explore whether and how these PCOS-SNPs may contribute to the pathophysiology of OCD.

In addition, future work should explore how our results may help explain the potential association of OCD with certain epigenetic changes, including in DNA methylation and miRNA profiles (Kandemir et al., 2015; Privitera et al., 2015; Nissen et al., 2016; Yue et al., 2016). The PCOS SNP rs1784692 in the present study lies in an intron of the protein “zinc finger and BTB domain containing 16” (ZBTB16), which can alter histone modifications and DNA methylation (Puszyk et al., 2013). Studies should examine whether and how ZBTB16 participates in OCD.

Our results may identify PCOS-related genes unique to OCD, given that we found a significant association of PCOS with OCD, but not with four other psychiatric disorders – even though all five disorders show considerable genetic overlap (6,786,993 SNPs) (Cross-Disorder Group of the Psychiatric Genomics Consortium, 2019). For example, OCD shows genetic correlation (r_g_) of 0.22 with BIP, 0.21 with MDD, and 0.35 with SCZ (Cross-Disorder Group of the Psychiatric Genomics Consortium, 2019). One study estimated that about 37% of genes are unique to OCD, and that such genes are enriched in neuroactive ligand-receptor interactions and signaling pathways involving G protein-coupled receptors (O'Connell et al., 2018). Among our PCOS-IVs, rs11031005 lies in an intergenic region of the gene encoding subunit beta of follicle-stimulating hormone, whose receptor is a classical G protein-coupled receptor expressed in neurons. Knocking down this receptor induces anxiety- or depression-like behaviors in mice (Bi et al., 2020). Future studies should explore the roles of follicle-stimulating hormone and its receptor in OCD.

Our results suggest that the positive associations of PCOS with AD (OR 2.75, 95% CI 2.10–3.60), BIP (OR 1.78, 95% CI 1.43–2.23), MDD (OR 2.79, 95% CI 2.23–3.50), and SCZ (OR 1.36, 95% CI 1.12–1.70) reported in observational studies (Cesta et al., 2016; Brutocao et al., 2018) may not reflect causality. One possible explanation is that residual confounders, rather than PCOS itself, increase the risk of psychiatric disorders. These confounders include high testosterone level, obesity (high body mass index), insulin resistance, and inflammation. Prenatal exposure to dihydrotestosterone can induce anxiety- and autism-like behaviors in rats (Domonkos et al., 2018; Xiang et al., 2020). Testosterone is elevated in the cerebrospinal fluid of schizophrenia patients (Misiak et al., 2018), and the androgen receptor is up-regulated in patients with bipolar disorder (Owens et al., 2019). Obesity increases risk of anxiety and depression in children and adolescents (Lindberg et al., 2020), and higher body mass index increases risk of attention-deficit/hyperactivity disorder (Martins-Silva et al., 2019). Insulin resistance has been positively associated with BIP, MDD, SCZ, and cognitive defects (Kullmann et al., 2016; Agarwal et al., 2020; Cuperfain et al., 2020; Zou et al., 2020), reflecting roles of insulin in energy metabolism and dopamine release in the central nervous system, contributing to memory and learning. Several inflammation-related factors have been recognized as biomarkers of psychiatric disorders (Yuan et al., 2019). Co-occurrence of OCD with other lifetime psychiatric disorders, which occurs in as many as 90% of patients with OCD (Stein et al., 2019), may also contribute to confounding. Stress can be another confounder: for example, stress due to such PCOS symptoms as hirsutism, acne, and infertility may make patients manifest anxiety- or depression-like symptoms.

Our findings should be interpreted with caution in light of several limitations. First, our IVs were derived from the largest European PCOS GWAS, which applied diverse diagnostic criteria: 51.4% of patients were diagnosed based on *ad hoc*, study-specific criteria, while 34.0% were diagnosed based on the Rotterdam criteria and 14.6% based on US National Institutes of Health criteria (Day et al., 2018; Zhu et al., 2020). This would increase the clinical heterogeneity of the patients. Second, there were relatively small numbers of OCD patients (2,088) and controls (7,037) in our sample. Third, all exposure and outcome data came from European populations, which may make our findings less generalizable to other ethnic groups or geographic areas.

Despite these limitations, our analysis suggests that, based solely on genetic factors, PCOS is a potentially causal factor for OCD, but not AD, BIP, MDD, or SCZ, in European populations. Appropriate screening for OCD, based on questionnaires or interviews, may be useful for European women with PCOS, which might enable early interventions involving a multidisciplinary approach (ZareMobini et al., 2018).

## Data Availability Statement

The datasets presented in this study can be found in online repositories. The names of the repository/repositories and accession number(s) can be found in the article/Supplementary Material.

## Ethics Statement

Ethical review and approval was not required for the study on human participants in accordance with the local legislation and institutional requirements. Written informed consent for participation was not required for this study in accordance with the national legislation and the institutional requirements.

## Author Contributions

LJ designed the study. LJ, JY, YC, and HP acquired and analyzed the data. LJ and JY drafted the manuscript. JS and HH obtained funding and revised the manuscript. All authors reviewed, contributed to, and approved the final manuscript.

## Conflict of Interest

The authors declare that the research was conducted in the absence of any commercial or financial relationships that could be construed as a potential conflict of interest.
